# Antimicrobial Effect of *Lippia citriodora* Extract in Combination with Gallic Acid or Octyl Gallate on Bacteria from Meat

**DOI:** 10.3390/foods13111643

**Published:** 2024-05-24

**Authors:** Javier Rúa, Javier Sanz-Gómez, Sheila Maestro, Irma Caro, María Rosario García-Armesto

**Affiliations:** 1 Department of Molecular Biology, University of León, 24007 León, Spain; javier.rua@unileon.es (J.R.);; 2 Institute of Food Science and Technology (ICTAL), La Serna 58, 24007 León, Spain; jjsang@unileon.es; 3 ALINS—Food Nutrition and Safety Investigation Group, University of León, 24007 León, Spain; 4 Area of Nutrition and Food Science, Faculty of Medicine, University of Valladolid, 47005 Valladolid, Spain; 5 Department of Food Hygiene and Technology, University of León, 24007 León, Spain

**Keywords:** phenolic compounds, *Lippia citriodora* extract, antimicrobial activity, poultry meat, modified atmosphere, refrigeration

## Abstract

Chicken meat and its derivatives are easily alterable. They are a nutritionally healthy food, and their consumption has seen a remarkable increase worldwide in recent years. At the same time, consumer demand for the use of natural products to control microbial growth is increasing. In this context, the antimicrobial capacity of a commercial extract of the lemon verbena (*Lippia citriodora*) plant, (LCE) was tested in binary combination with gallic acid or octyl gallate against two strains of lactic acid bacteria (LAB) of meat origin: *Carnobacterium divergens* ATCC 35677 and *Leuconostoc carnosum* ATCC 49367. First, the antimicrobial potential was evaluated by the checkerboard microdilution method at the optimal growth temperature of each and at 4 °C, pH 5.7 and 6.7, in culture medium. Octyl gallate was the most effective antimicrobial against the two bacteria under all study conditions. At 4 °C, the combination of LCE with octyl gallate had a similar antimicrobial effect on the two LAB, being bactericidal at pH 6.7. In chicken breast, this effective combination was tested in normal or modified atmosphere and refrigerated (4–8 °C) for 9 days. LCE + OG in modified atmosphere reduced the different microbial groups studied, including the lactic acid bacteria as the main microorganisms responsible for the spoilage of fresh meat. Further research could pave the way for the development of novel strategies contributing to the technological stability, security, and functional properties of chicken meat.

## 1. Introduction

Chicken meat is a nutritionally healthy food that has an affordable price, which is why its consumption has experienced a notable increase worldwide in recent years [1]. However, with increasing production and consumption, a significant percentage of poultry meat is lost each year due to spoilage. Spoilage of meat can be attributed to prolonged storage times, inadequate storage temperatures, contamination, or high pH levels. In addition, spoilage is detrimental to product quality due to off-flavours, off-odours, and microbial growth.

Microbial contamination present in fresh meat occurs in most cases in surface areas of the meat, or adjacent tissues, such as skin or parts of the slaughtered animal, that have been contaminated and come into contact with the meat during processing operations. The sources of microbial contamination are diverse, including the production environment, handlers, surfaces, and/or utensils, etc.

Lactic acid bacteria are common causes of the reduced shelf life of perishable food products. However, the status of several species belonging to this group (comprising more than 10 genera) is highly controversial in relation to their role in meat spoilage [2]. In fact, some of these species are considered as biopreservatives of meat products [3]. In this study, strains of the species *Carnobacterium divergens* and *Leuconostoc carnosum* were used as target microorganisms of the two PCs and the lemon verbena extract mentioned above.

*Carnobacterium divergens* has been associated with the spoilage of foods and meat products [4]. They are Gram-positive, immobile, non-spore-forming, facultative anaerobic bacteria. They appear alone, in pairs, or in short chains. Colonies on solid medium are creamy-white, circular, convex, and shiny, varying in size from 0.5 to 1.5 mm. Strains of this species are mesophilic psychrotolerant: they grow at temperature intervals of 0–40 °C and do not grow at 45 °C. They also do not grow at pH below 4.5, and they exert heterofermentative activity, producing lactic acid, CO_2_, ethanol, and acetate from glucose fermentation.

In general, *Carnobacterium* spp. is important in the alteration of meats stored under vacuum or in modified atmospheres [5,6]. Its growth in these products is favoured by its tolerance to microaerophilic conditions and low pH values [7,8]. In addition, the genus is among the spoilage microorganisms of processed meat products, obtained from whole or minced muscle parts or from their mixtures with animal fats or vegetable oils [9].

*Leuconostoc* is one of the LAB spoilers of cooked and vacuum-packed meat products [8,10]. This genus is defined as Gram-positive, non-motile, non-spore-forming, and catalase-negative bacteria. Morphologically, it is considered to be cocci or coccobacillary. *Leuconostoc* species can be psychrotrophic, and the optimum growth temperature is between 20 and 30 °C. They are not normally acidophilic and prefer an initial medium pH of 6–7. They are heterofermentative and metabolize glucose-producing lactic acid, ethanol, and acetic acid. *Leuconostoc carnosum* is the species used in this study and is characterized by lenticular-shaped cells; colonies on MRS agar are small, smooth, round, and greyish white. Growth occurs at 10 °C, and most do not grow at 37 °C.

While techniques such as heat treatment, salting, and acidification have been applied in the food industry for decades to minimize spoilage, the possibility of using polyphenolic compounds or natural extract as an alternative emerging technology to increase the shelf life of meat and poultry products is a growing demand. In order to increase its conservation period, different procedures have been tested in recent years [11,12], among which are those based on “barrier technology” that allows the design of innovative food products with a greater guarantee of stability against microbial and oxidative alteration, as well as greater functional quality. One possibility included in this group of technologies is the application of several antimicrobial chemical agents in combination, as ingredients of chicken meat derivatives (patties, sausages, etc.) or as solutions used externally (breast, thighs, etc.). Additionally, these ingredients could have antioxidant and functional properties.

Among the compounds with antimicrobial capacity found naturally in some foods (for example, fruits, spices, etc.) and/or that can be added voluntarily to them are various phenolic compounds (PCs). This term is used to designate substances that have an aromatic ring carrying one or more hydroxyl substituents, including their functional derivatives (esters, glycosides, etc.) [13,14]. PCs are therefore components of plant essential oils that are added in a wide variety of food systems [15] and can be used as potential barriers to control pathogenic and/or food disrupting microorganisms [16]. They can be used individually or in various combinations with other antimicrobial agents against the target (spoilage and/or pathogenic) bacteria.

PCs can be classified into two large groups according to their origin: natural or synthetic. In this study, a natural (gallic acid) and a synthetic derivative (octyl gallate) PC were used in addition to commercial extract of the lemon verbena (*Lippia citriodora*) plant (LCE).

LCE contains PCs with antioxidant and anti-inflammatory capacity. Its composition includes glycosylated phenylpropanoids such as verbascoside (which is the most abundant, representing 25% of total PCs in the extract), eukovoside, and martinoside, as well as flavonoids (luteolin, apigenin, and chrysoeriol, among others), conjugated with two diglucuronide molecules [17]. Studies carried out by the Institute of Molecular and Cellular Biology of the Miguel Hernández University (Elche) on this LCE have revealed some beneficial health effects in experimental animals and/or in humans: it helps to reduce body fat levels and maintain a balanced weight by acting on the enzymes responsible for lipid metabolism. Likewise, it enhances endogenous antioxidant defences, significantly increasing glutathione reductase levels in blood cells [18,19]. Recently, it has also been shown that the incorporation of antioxidant supplements based on lemon verbena extracts could be considered as an alternative therapy in the treatment of multiple sclerosis in humans [20]. Regarding the control of microorganisms, LCE has been found to be more effective as an antimicrobial for Gram-positive (*Bacillus cereus* and *Staphylococcus aureus*) than Gram-negative bacteria (*Escherichia coli* and *Salmonella enterica*).

The gallic acid (3,4,5-trihydroxy-benzoic acid, C_7_H_6_O_5_) (GA) used in this study can be found in foods in free form or as part of tannins (mainly, in red wine, tea leaves, and vegetable galls). It is soluble in ethanol up to a final concentration of 0.16 g/mL and is included in the database of flavouring substances authorised in the European Community, under reference no. FL: 08.080 [21]. It has antibacterial, antiviral, analgesic, and antiapoptotic activity [22].

A synthetic derivative of gallic acid, octyl gallate (octyl 3,4,5-trihydroxybenzoate, C_15_H_22_O_5_) (OG) has also been used in this study. It is lipid soluble and heat stable. Like all gallates, it can effectively chelate metal ions, retarding metal ion catalysed lipid oxidation. Its use as an antioxidant has been approved in foods in the European Union [23] and the United States [24].

The general objective of this study was to evaluate the antibacterial activity, mainly the spoilage population, of a natural extract and other PCs to extending the shelf life of fresh chicken meat. The first objective was to verify the antimicrobial capacity of GA or OG in binary combination with LCE against both strains of *C. divergens* and *Le. carnosum*. Secondly, we used the binary combination of the most effective PC with LCE to control the deterioration of poultry in a modified aerobic atmosphere under refrigeration for 9 days.

## 2. Material and Methods

### 2.1. Antimicrobial Activity of LCE, Gallic Acid, and Octyl Gallate in Broth under Different Temperature and pH Conditions

#### 2.1.1. Analysis of Composition of Lemon Verbena Commercial Extract

The commercial lemon verbena extract used in this study was, according to the information provided by the commercial company, composed mainly of verbascoside, with much less isoverbascoside and other phenylpropanoids. This is in agreement with the previously mentioned work by Quirantes-Piné et al. (2009) [25].

In addition, we carried out an HPLC-MS analysis of the product batch used in this work, according to the method described in the Appendix A, using 5 μL of a solution of LCE (5% *w*/*v* in methanol) in 100 mL MeOH. The HPLC equipment was a Bruker Elute UHPLC, and the mass spectrometer was a Bruker “tims-TOF” time-of-flight mass spectrometer with ion mobility (although no mobility was used). The compounds identified were mostly verbascoside at lower concentrations (<0.5 mg/L), quercetine, phenyl caffeate, galangine, and chrysine, as well as sakuranetine, luteolin, and kaempherol at much lower concentrations.

#### 2.1.2. Microorganisms, Maintenance, and Culture

Two lactic acid bacteria (LAB) were used in this study: *Carnobacterium divergens* ATTC 35677 (originating from vacuum-packed minced beef) and *Leuconosto ccarnosum* ATCC 49367 (originating from chill-stored meat), having been purchased from the American Type Culture Collection (Manassas, VA, USA). Stock cultures of each bacterial strain were maintained in Eppendorf tubes containing Tryptic Soy Broth (TSA) (Oxoid Ltd., Basingtoke, UK) in the presence of 50% (*v*/*v*) glycerol at −80 °C. Frozen stock cultures were activated by transferring 20 μL into 4 mL of Tryptic Soy Broth + 0.6% yeast extract (*w*/*v*) (Oxoid Ltd.) (TSBYE) and incubating for 24 h at 30 °C ± 1 °C for *C. divergens* or 48 h at 25 °C ± 1 °C for *Le. carnosum*.

#### 2.1.3. Chemicals and Preparation of Stock Solution

Gallic acid (GA) 99% purity HPLC and octyl gallate (OG) 99% purity HPLC were obtained from Sigma-Aldrich (St. Louis, MI, USA). *Lemon verbena* commercial extract (PLX^TM^) (LCE) (25% verbascoside) was kindly provided by Monteloeder S. L. (Elche, Spain). Stock solution of the phenolic compounds (PCs) used (60 mg/mL for GA and 0.8 mg/mL for OG) were freshly prepared by dissolving the appropriate amount of the PC in 2 or 4 parts of 95% (*v*/*v*) ethanol and 6 or 8 parts of culture medium used in the experiment: LSM Broth, consisting of Iso-Sensitest Broth (ISB, 90%) plus the Man, Rogosa and Sharpe Broth (MRSB) (10%) with pH adjusted to 6.7 [26] or 5.7. The PLXTM stock solution (10 mg/mL in 40% ethanol) was stored frozen.

#### 2.1.4. Minimal Inhibitory Concentration and Antimicrobial Interaction Testing

Minimal inhibitory concentration (MIC) determination and antimicrobial interaction testing were performed with the checkerboard method on microtiter plates with LSM. The methodology to carry out the antimicrobial assay to estimate the MIC values of the phenolic compounds and LCE is described in ISO Standard 20776-1:2006 [27]. Briefly, after checking the recovery ability and purity of strains, inoculum for the anti-microbial assays was prepared by diluting the overnight cultures in TSBYE grown as explained in Section 2.1 and then with LSM (24 h a 30 °C for *C. divergens* and 48 h at 25 °C for *Le. carnosum*) to obtain a concentration of 5 × 10^5^ cfu/mL. The concentration of microorganisms was checked by the Miles and Misra technique [28]. The checkerboard method was performed as described by Gutiérrez-Fernández et al. [29]. The MIC of each compound alone or in combination was defined as the minimal concentration of anti-microbial compound that inhibits visible growth of the strain tested [27]. The incubation of the microtiter plates was carried out aerobically. Each strain was incubated at its optimum growth temperature (30 °C for *C. divergens* and 25 °C for *Le. carnosum*) and both at 4 °C (meat refrigeration temperature). The pH values used were 5.7 and 6.7.

Growth in each well was quantified using a visual observation method, and turbidity was interpreted as visible growth of bacteria. At least two independent tests were performed in duplicate for each strain and binary combination under each condition (pH and temperature).

Individual MICs of each compound were estimated in the same microtiter plate from the data of the column or the row in which one of the compounds was absent. MIC data were transformed to fractional inhibitory concentration (FIC). The FIC of an individual antimicrobial compound was the ratio of the concentration of the antimicrobial at the inhibitory concentration, with a second compound to the concentration of the anti-microbial by itself as follows:

FICA = MIC of A with B/MIC of A

FICB = MIC of B with A/MIC of B

The FIC index (FICI) was calculated as follows, with the FICs for the individual antimicrobials:

FICI = FICA + FICB

The criteria used to determine the type of combined antimicrobial effect were synergy, FICI ≤ 0.5; no interaction, FICI > 0.5–≤ 4.0; and antagonism, FICI > 4.0 [30].

#### 2.1.5. Determination of Bacterial Growth and Interactive Effects

The growth analysis of two LAB under the above-mentioned pH conditions and temperature was performed by taking samples from the wells of the microtiter plates corresponding to the MIC and 2 MIC of each individual PC/LCE and those corresponding to the sums of MIC of the two antioxidants (MICA + MICB) and double the MICs (2MICA + 2MICB).

To count the microbial load, the miniaturized method known as the “surface drop” technique was used, following the recommendations of the International Commission on Microbiological Specifications for Foods [31]. Aliquots of 20 μL were removed from each culture well at 0 and 24 h and diluted in 180 μL of bacteriological peptone (0.1%, *w*/*v*; Oxoid Ltd.). These suspensions were then serially diluted using 10-fold dilutions, with 20 μL aliquots plated onto Trypticase soy agar (TSA) (Conda Pronadisa, Madrid, Spain), incubated for 24 or 48 h at 30 or 25 °C, and counted for survival estimation. Plates showing 1–50 cfu were counted, and results are shown as log_10_ cfu per millilitre. At least two trials on different days were carried out in duplicate for each strain and treatment, and the results presented are the mean of all of them. This number is acceptable for statistical treatment. However, when the results were not clear enough, a greater number of replicates were analysed.

A bactericidal and bacteriostatic effect were, respectively, defined as ≥3 log_10_ or <3 log_10_ reduction in colony counts at 24 h (*C. divergens*) or 48 h (*Le. carnosum*) compared with the starting inoculum. Synergism was defined as a decrease in a viable count of ≥2 log_10_ cfu/mL, or indifference as a decrease in viable count of <2 log_10_ cfu/mL, and antagonism as an increase in viable count of ≥2 log_10_ cfu/mL of the combination com-pared with the most active single PC after 24 or 48 h [32].

### 2.2. Effect of LCE and Octyl Gallate on the Shelf Life of Poultry Fillets in a Normal or Modified Atmosphere under Refrigeration

#### 2.2.1. Experimental Design

The boneless, skinless raw chicken breast fillets (*Pectoralis major*) were purchased from a supermarket and then placed in plastic bags and transported in isothermal packaging to the laboratory of the Institute of Food Science and Technology (ICTA, ULE, León, Spain). Breast fillet halves were used in batch L1 and the other halves in batch L2. Every batch contained four samples: two without and two with OG (25 µg/mL) + LCE (2500 µg/mL). Each 25 cm^2^ sample was placed in a sterile Petri dish and stored at 4 °C for 30 min before adding OG + LCE. Moreover, 200 μL of OG-LCE (25–2500 µg/mL) was added to the 25 cm^2^ breast fillet samples, which were then stored for one hour at 4 °C before analysis or packaging.

#### 2.2.2. Samples Packaging

After cooling, one sample with OG + LCE was packed into a modified atmosphere (MA) (30% CO_2_ and 70% N_2_), and the other into a normal atmosphere (NA). The samples without the combination were also placed under the conditions mentioned before. For MA treatment, a single sample of 25 cm^2^ was packed using a 20/100 polyamide/polyethylene bag (IndustriasPargon, Salamanca, Spain, with a thickness of 120 µm and oxygen permeability of 50 cm^3^/m^3^ × bar × 24 h at 23 °C), during 2 min per packaging bag and maintained under <10 °C. For NA treatment, individual samples of 25 cm^2^ were placed in sterile Stomacher bags without heat-sealing. All packaged samples were stored at 4 °C for six days and at 8 °C for three additional days. The samples were analysed at days 0 (one hour after inoculation and stored at 4 °C), 2, 4, 6, and 9 of the conservation-storage periods.

#### 2.2.3. Microbiological Counts

Psychrotrophic (PSC), *Pseudomonas* (PSEUDO), *Enterobacteriaceae*, *E. coli*, Aerobic plate Count (PAC), LAB, sulphite-reducing clostridia bacteria, and *Staphylococcus aureus* were counted. Each 25 cm^2^ (c.a. 10 g ± 0.1 g) sample was aseptically placed in a sterile Stomacher bag with 90 mL of sterilized peptone water (0.1%: Oxoid Ltd., Hampshire, UK) and homogenized for 2 min with a Stomacher-400 circulator (Seward, West Sussex, UK). Appropriate serial decimal dilutions were prepared, plated on relevant media in duplicate (Oxoid Ltd., Basingstoke, Hampshire, UK), and incubated as follows: psychrotrophic bacteria were determined on Plate Count Agar at 7 °C for 10 days [33]; *Pseudomonas* spp. were counted on *Pseudomonas* agar base supplemented with cetrimide, fucidin, and cephalosporin (SR0103; Thermo Fisher: Waltham, MA, USA) at 7 °C for 48 h [34]; total *Enterobacteriaceae* counts were performed on Violet Red Bile Glucose Agar at 37 °C for 24 h [35]; *E. coli* were plated on Tryptone Bile Glucuronide agar (TBX agar) incubated at 37 °C for 24 h [36]; total viable aerobic bacteria were plated on Plate Agar Counts at 30 °C for 72 h [33]; LAB counts were determined on de Man Rogosa Sharpe Agar (MRSA) according to ISO 15214:1998 [37] at 30 °C; *Staphylococcus aureus* were plated on Baird Parker Agar [38] at 37 °C for 24 h, and colonies with characteristic morphology were confirmed using a Staphylase test (Oxoid: DR0595); and sulfite-reducing clostridia bacteria were enumerated on TryptoseSulfiteCycloserine agar at 37 °C for 24 h [39].

### 2.3. Statistical Analysis

All experiments were performed using a minimum of two trials in duplicate. The results are expressed as mean ± standard deviation (SD). Microbiological results were transformed into logarithms of the number of colony-forming units (cfu) using Excel software (Microsoft 365). Statistical analyses were performed with the SPPSS statistics program (Version 26, IBM, New York, NY, USA). An analysis of variance, with a post hoc Tukey test, was conducted to determine any significant differences between means, and principal component analysis (PCA) was used to extract the components with the highest explanatory power for the data set. In all cases, the level of statistical significance was *p* < 0.05.

## 3. Results and Discussion

### 3.1. Antimicrobial Activity of LCE, Gallic Acid, and Octyl Gallate in Broth under Different Temperature and pH Conditions

The antimicrobial activity of the binary combinations of LCE with GA or OG against *C. divergens* and *Le. carnosum* was determined by the checkerboard microdilution method, at two pH values (5.7 and 6.7) in LSM medium. These two pH values were chosen because that of chicken breast is between 5.7 and 5.9, and that of chicken thigh is between 6.4 and 6.7 [40]. The study was carried out at optimal growth temperatures (30 °C for 24 h for *C. divergens* and 25 °C for 48 h for *Le. carnosum*) and at 4 °C for 14 days for both strains.

Individual MIC (minimal inhibitory concentration) values for each of the antimicrobials used were obtained from the microtiter plates. Table 1 shows the mean values with the standard deviations of these MICs under the indicated conditions. In general, the values were higher (close to two orders of magnitude) for GA and LCE compared to OG (synthetic phenolic compound) under all the tested conditions and for both bacteria. In addition, they were very similar for GA and LCE, with no significant differences between them for either pH or in either bacterium. An increase in octyl gallate MICs was only detected at pH 6.7 for *C. divergens*. Temperature did not seem to influence OG and LCE MICs in either bacterium, since values were similar at optimal and refrigerated temperatures (4 °C), although the latter was obtained after 14 days’ storage.

The results of the interaction of the antimicrobial compounds in binary combination (FIC, fractional inhibitory concentration, and FICI, fractional inhibitory concentration index, values) are shown in Table 2 for the two bacteria under optimal growth conditions. None of the combinations tested (different bacteria and combination of antimicrobials) showed an antagonistic effect for the two pH values. As can be seen, the FICA (fractional inhibitory concentration of LCE) and FICB (fractional inhibitory concentration of GA or OG) values were less than 1 for both bacteria and under all conditions.

In all binary combinations tested, LCE MICs decreased by approximately two-thirds compared to the corresponding LCE alone MIC values, for both bacteria and at both pH values. The MIC values for GA and OG, under all conditions and for both bacteria decreased by approximately one-third compared to the corresponding GA or OG alone MIC values. This decrease in MIC values means that OG (synthetic PC) was less present in the combination (or would be needed in a smaller quantity in the combination with LCE for the antimicrobial effect to occur). The concentration of GA, which had the highest MICs, also decreased. In addition, the lower decrease exerted on LCE by the two PCs allowed this extract to be maintained to exert its beneficial properties. Therefore, LCE used in combination with these two antimicrobials as food additives could obtain beneficial properties and extend the useful life of the product. The FICI is the mathematical expression that measures the effect of the interaction in a binary combination of antimicrobials. Several criteria have been described to interpret the results of the interaction of antimicrobial agents. According to those used in the present study, we found no interaction (FICI > 0.5–≤4.0), which occurs when two antimicrobials in combination give a result equivalent to the sum of each antimicrobial acting independently [41].

The effect of chilling temperature (4 °C for 14 days) on the antimicrobial activity of the combination occurred with the binary combination of LCE with octylgalactate, with the latter being the most effective antimicrobial (lower MIC). As can be seen in Table 3, the refrigeration temperature had no effect on antimicrobial activity in this binary combination compared to the results obtained at optimal growth temperatures, producing a similar decrease in MIC values in the combination under both conditions (Table 2 and Table 3). As we previously indicated, these additives could be applied to foods maintained under refrigeration for 14 days to control the growth of the two bacteria.

In previous studies performed by our research group [42], we obtained similar LCE MIC values in other LAB from the genera *Lactobacillus*, *Lactococcus*, and *Streptococcus*, grown at 35 °C and pH 6.7 (2500–4500 μg/mL), but we obtained higher values (7500 μg/mL) for these genera at pH 5.5 and 35 °C. Values at 4 °C (78–156.26 μg/mL at pH 6.7 and 78–1875 μg/mL at pH 5.5) were lower than those obtained in this study.

A possible explanation for the different behaviour of the strains at the pH values studied is that the antimicrobial compounds present in LCE (mainly verbascoside and other phenolic compounds such as quercetin, phenyl caffeate, galangine, and chrysin in much lower concentration) could show a different degree of dissociation at both pHs. However, when we calculated the percentages of verbascoside at pH 5.7 and 6.7, we found that they were almost entirely undissociated (99.95% at pH 5.7 and 99.51% at pH 6.7), considering a predictive pK_a_ value of 9.01 for verbascoside, as an experimental pK_a_ value is not available [43]. In this sense, other authors [44] described that the MIC values for the undissociated form of a weak acid, such as lactic acid, were higher at pH 5.7 than at pH 6.6, with the difference for *Lactobacillus* M18 at 20 °C being 7.8-fold. In general, undissociated forms of weak acids are reported to be more effective antimicrobials than dissociated ones. In any case, in the present study, we did not observe differences in MIC values at the two pHs used.

The antimicrobial activity of verbascoside against *S. aureus* has been attributed to an inhibition of leucine uptake [45]. On the other hand, verbascoside contains several hydroxyl groups in its structure, which can establish hydrogen bonds with the polar head of cell membrane phospholipids. As the phosphate groups of the polar head of the phospholipid need to be ionized to establish such interactions with verbascoside, there would be fewer membrane-disrupting effects at lower pHs [46].

Regarding OG, the existence of a C8 alkyl chain produced a decrease in the MIC of approximately 160 times at pH 5.7 for both bacteria, 100 times for *C. divergens*, and 140 times for *Le. carnosum* at pH 6.7, in relation to gallic acid. It has been established that the antimicrobial activity of alkyl gallates increases as the chain length increases, which may account for the greater antimicrobial effectiveness of octyl gallate (for a review, see Takai et al. [47]).

In the reviewed literature, we found MIC values for OG between 6 and 50 μg/mL [29,48,49,50], similar to those we obtained for the two bacteria. On the other hand, Król et al. [51] proposed that the rupture of the membrane and inhibition of the FtsZ protein, a cytoplasmic protein involved in cell division, contributes to the antibacterial activity of alkyl gallates.

Similar MIC values (between 2900 and 4600 μg/mL) have been described for GA against the following LAB: six strains of *Enterococcus faecalis* [29], one of dairy origin and another ATCC 29212 [52], and two from *Lactobacillus*: *L. plantarum* and *L. hammesii* [53]. However, values equal to or greater than 8000 μg/mL were described for various species of *Lactobacillus* [54] and much lower than 200–300 μg/mL for others [55]. These low MIC values may be due to the fact that the detection method used was not the microdilution method employed in this study, or to other factors, such as the different growth stage of the inoculum, the quantification method, etc.

In general, we did not find a pH effect in any of the conditions studied regarding the antimicrobial activity of GA and OG. Gallic acid is a weak phenolic acid (pKa ≈ 4.0, [56]), and at pHs 5.7 and 6.7, the amount of the undissociated form was very low (0.2% at pH 5.7 and 0.02% at pH 6.7), which could account for the similar antimicrobial activity. It has been established that the antimicrobial activity of weak phenolic acids is dependent on the pH and the concentration of the undissociated form. This undissociated form crosses the cell membrane by passive diffusion, altering its structure and possibly acidifying the cytoplasm and causing protein denaturation [5,57,58,59].

### 3.2. Effect of pH and Temperature on the Antimicrobial Activity

The checkerboard method simply reflects the bacteriostatic effect, while the study of bacterial growth allows us to determine both the bacteriostatic and bactericidal activity of a compound. For this reason, we determined the growth of *C. divergens* (at 0 and 24 h) and *Le. carnosum* (at 0 and 48 h) without antimicrobials or with the binary combinations used in this study, at MIC values or 2 MIC of each compound and under the conditions studied. This enabled us to determine the antimicrobial activity of each compound as well as the effect of interactions between them (Table 4 and Table 5). Under all the conditions tested, the growth of the two bacteria in LSM medium was similar in the absence of the compounds used.

It was observed that GA seems to be a bacteriostatic agent for both *C. divergens* and *Le. carnosum* since <3 log_10_ reductions were produced in the cfu count with regard to the initial inoculum. As can be seen in Table 4 (under optimal growth conditions), a decrease in viable counts was generally observed with respect to the initial inoculum for *Le. carnosum* and at the two pHs; however, for *C. divergens*, the log cfu/mL were slightly lower or higher than the initial inoculum, and growth was always lower than that of the control. In general, OG was bacteriostatic (<3 log_10_ reductions in cfu/mL) for *C. divergens* and at both pHs. However, for *Le. carnosum*, it was bactericidal (reductions ≥3 log_10_ of cfu/mL compared to the initial inoculum) at both pH values.

For *C. divergens*, and at MIC values, antimicrobial combinations showed an additive effect (decrease in viable counts of <2 log_10_ cfu/mL compared to the most active antimicrobial agent alone after 24 h) for pH 5.7. However, at pH 6.7, the GA + LCE combination showed a synergistic effect (decrease in the number of viable colonies of ≥2 log_10_ cfu/mL), and the OG + LCE combination showed an additive effect. For this bacterium, and at values of 2 MIC of the combined antimicrobials, a synergistic effect was observed for GA + LCE at both pH values, while for OG + LCE, the effect was additive.

The GA + LCE and OG + LCE combinations at MIC values in *Le. carnosum* showed a synergistic and additive effect, respectively, for both pHs. The GA + LCE combination at concentrations of 2 MIC and pH 5.7 showed an additive effect. However, at pH 6.7, it was not possible to detect a hypothetical synergistic effect in combination with LCE, but instead an additive or non-interaction effect, since gallic acid at this concentration (2 MIC) had a bactericidal effect. This effect was also observed for the OG + LCE combination at concentrations of 2 MIC and at both pH values (Table 4).

Another study was carried out at 4 °C after 14 days (Table 5) using the most effective combination of antimicrobials (OG + LCE) and the individual antimicrobials. At MIC and 2 MIC, OG had a bacteriostatic effect at pH 5.7 for both bacteria. However, at pH 6.7, the effect was bactericidal at concentrations of 2 MIC for both bacteria. LCE did not seem to exert any effect individually, since growth was observed at the two concentrations tested.

Regarding the combination, an additive effect could be seen for both bacteria at pH 5.7 and at MIC concentrations, whereas a synergistic effect was observed for pH 6.7 at the same MIC concentrations. This could lead one to think that at pH close to neutrality, the combination contributes to greater antimicrobial activity under refrigerated conditions. At concentrations of 2 MIC, the OG + LCE combination showed a synergistic effect for both bacteria at pH 5.7. At the other pH value, the combination could have an additive effect, since, individually, OG is bactericidal.

### 3.3. Effect of LCE and Octyl Gallate on the Shelf Life of Poultry Fillets in a Normal or Modified Atmosphere under Refrigeration

Table 6 shows the effect of the aerobic (normal, NA) or modified atmosphere (MA) and treatment (OG + LCE) for the microbial groups studied during the 9 days of storage of chicken breast: 0–6 days at 4 °C and a further 3 days (up to 9 days) at 8 °C. A statistical analysis of each of the microbial groups was carried out to evaluate the effects of the variables throughout the storage time.

On the day the test started (time zero: one hour after inoculation and stored at 4 °C), the highest microbial load in chicken breast was detected for MRS, PCA and PSC microbial groups (around 5 log_10_ cfu/mL), while the lowest microbial load was for TSC (approximately 1 log_10_ cfu/mL). Throughout storage, an increase in the microbial load was observed for PSC in general for any atmosphere, with or without additives. On the other hand, a more evident increase in the microbial load was detected under normal atmosphere conditions (air) for MRS, PCA, PSC, PSEUDO, and VRBGA, with the increase for these groups being scarcely noticeable in a modified atmosphere, both with and without the addition of OG + LCE. The groups showing no or very little increase in microbial load under all conditions were BP and TSC, while for coliforms (TBX), even a slight decrease was observed (around 1 log_10_ cfu/mL) after of 9 days of storage.

The passage from 6 days (4 °C) to 9 days (8 °C) caused an increase in the microbial load under normal atmosphere conditions for MRS, PCA, PSC, PSEUDO, and VRBGA, while it decreased slightly for BP, coliforms, and TSC.

In general, throughout storage, microbial loads were lower in the modified atmosphere condition with and without additives. Although the additives (OG + LCE) did not seem to have any significant effect on decreasing the growth of microorganisms, the combination would at least provide the chicken breasts with an antioxidant environment and other organoleptic properties as well as microbiological safety.

**Figure 1:**
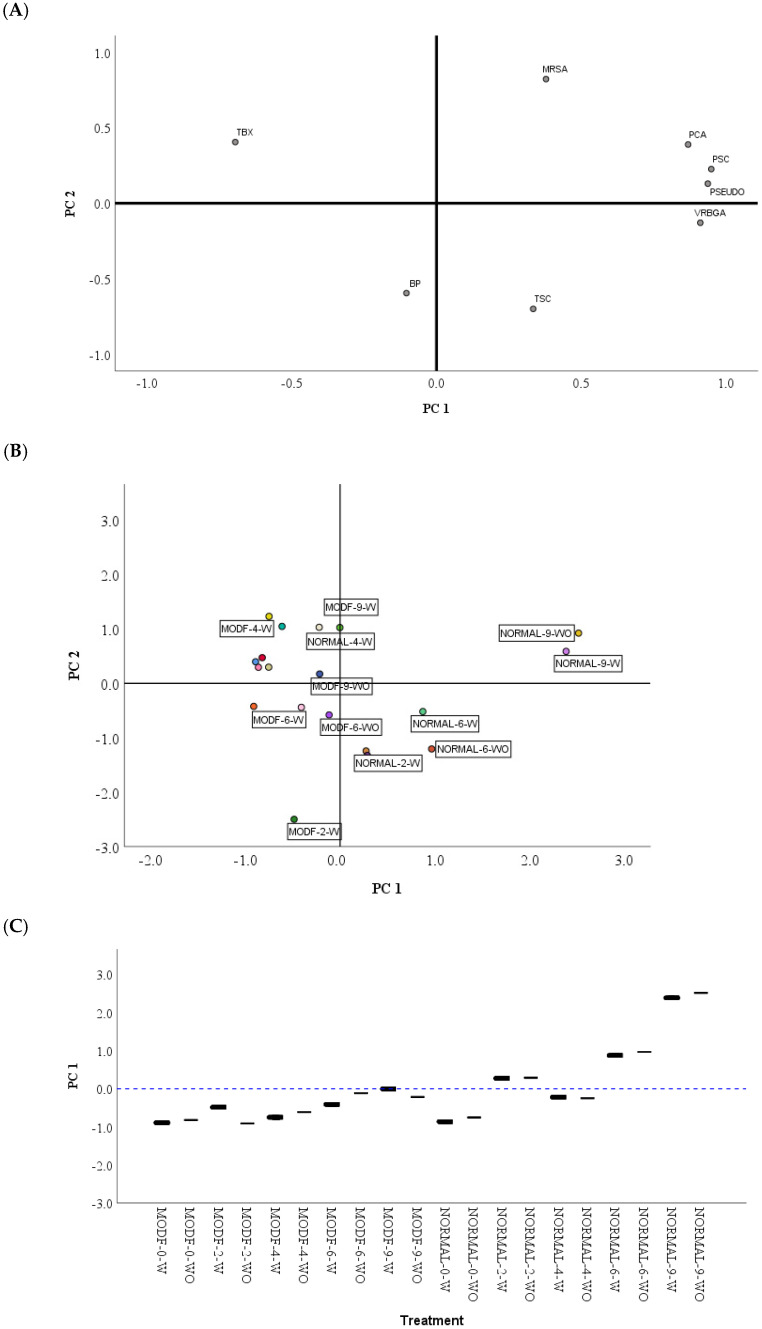
Principal component analysis: (**A**) Factor loadings and score plots; (**B**) by treatment type and (**C**) the distribution of different treatments according to PC 1 obtained from factor analysis using 8 selected groups of microorganism counts: BP, Baird Parker; MRS; lactic acid bacteria; PCA, aerobic plate count; PSC, psychotrophic bacteria; PSEUDO, *Pseudomonas* spp.; TBX, coliforms; TSC, sulfite-reducing CLOSTRIDIA; and VRBGA; *Enterobacteriaceae*.

Factorial analysis was carried out using the results of eight selected variables from poultry fillet samples with and without OG + LCE, stored in an aerobic (NA) and modified atmosphere (MA). Table 7 shows the component matrix and the two main components identified; both have eigenvalues >1 (first component PC1 = 4.2 and second component PCA 2 = 1.846), which satisfies the Kaiser Guttman criterion of selecting the principal component and, by this rule. The first two principal components were considered given the maximum explanation of variability [60]. Both conjunct PCs explained 79.5% of the variance from eight selected variables in NA and MA packed. The factor loading and score plots are shown in Figure 1A. PC1 explained 55.8% of the variance among microbial counts. This factor was highly positively associated with psychrotrophic bacteria and *Enterobacteriaceae* and slightly negatively associated with coliforms. PC 2 explained 23.7% of the variance between microbial count groups. This factor was positively influenced by MRS counts or lactic acid bacteria and negatively by clostridia sulphite reducing. Figure 1B shows that PC1 was able to separate samples according to the storage conditions from NA to MA treatment. NA showed the highest counts of psychrotrophic bacteria (10.22 ± 0.19 log_10_cfu/g) and *Enterobacteriaceae* (4.64 ± 0.18 log_10_cfu/g) and lower coliform counts (0.78 ± 0.14 log_10_cfu/g). It means that the main groups of microorganisms that spoil chicken breast in aerobic packaging were psychrotrophic microorganisms such as *Pseudomonas* spp. and psychrotrophic enterobacteria (see Figure 1C). On the contrary, samples stored on MA located on the central (MA 9-W) and the negative side of the graph showed fewer psychotrophic bacteria and total aerobic counts of 6.56 ± 0.53 and 6.53 ± 0.37 log_10_cfu/g, respectively. As depicted in Figure 1B, the scattering of the treatments with and without OG + LCE was gradually transformed over storage time, as well as the presence or lack of presence of this combination. Packaging treatment samples were moved towards the right along the PC2 axis. From NA packaging on the ninth day onwards to MA samples on the sixth day, the last samples contained lower MRSA counts. Moreover, MA treatments with OG + LCE on the second and sixth days were placed opposite the MRSA counts. LAB were the predominant spoilage microorganisms in refrigerated-stored fresh meat under MA generating off-flavours, slime, and discoloration [16]. So, the addition of OG + LCE combined with MA could extend the shelf life of chicken breast.

## 4. Conclusions

In culture conditions, the antibacterial activity of OG and its combination with LCE was higher at pH values close to neutrality under refrigeration. In chicken breast, this combination in a modified atmosphere reduced the different microbial groups studied, including lactic acid bacteria as the main microorganisms responsible for the spoilage of fresh meat.

Further research is warranted to elucidate the precise mechanisms of antimicrobial activity of this combination or others with natural extracts and phenolic compounds. This could pave the way for the development of novel strategies contributing to the security and functional properties of chicken meat.

## Figures and Tables

**Table 1:** Minimum inhibitory concentration (μg/mL) of the two PCs and LCE against two strains of LAB in LSM broth.

Bacteria Tested	Compound	Ph			
		5.7		6.7	
		Optimum t ^a^	4 °C	Optimum t ^a^	4 °C
*C. divergens* ATCC 3567					
	Gallic acid	3575.00 ± 1151.00 ^a^	ND	4000.00 ± 828.00 ^a^	ND
				
	Octyl gallate	22.63 ± 3.74 ^b^	25.00 ± 0.00 ^a^	37.90 ± 11.15 ^b^	50.00 ± 0.00 ^a^
				
	LCE	2916.00 ± 1707.00 ^a^	2500.00 ± 0.00 ^b^	3333.00 ± 1290.00 ^b^	5000.00 ± 0.00 ^b^
*Le. carnosum* ATCC 49367					
	Gallic acid	3716.00 ± 68.00 ^a^	ND	4139.00 ± 1517.00 ^a^	ND
				
	Octyl gallate	21.48 ± 7.03 ^b^	12.50 ± 0.00 ^a^	30.00 ± 18.95 ^b^	25.00 ± 0.00 ^a^
				
	LCE	4166.00 ± 1290.00 ^a^	2500.00 ± 0.00 ^b^	2916.00 ± 1020.00 ^a^	1250.00 ± 0.00 ^b^
				

Values are the means of at least two experiments in duplicate ± SD. ND: not determined. Optimum temperatures: 30 °C (*C. divergens* ATCC 3567) and 25 °C (*Le. carnosum* ATCC 49367). 24 h culture at 30 °C, 48 h culture at 25 °C, and 14 days culture at 4 °C. For each bacterium, different letters within a column mean significant differences (*p* < 0.05).

**Table 2:** Antimicrobial activities of binary combinations of LCE with GA or OG against two strains of LAB in LSM broth at optimum growth temperature for each strain.

Bacteria Tested	Binary Combination (A + B)	pH	MIC_A_ (Alone/Comb)	FIC_A_	MIC_B_ (Alone/Comb)	FIC_B_	FICI	Interpretation
*C. divergens* ATCC 35677								
	LCE + Gallic acid	5.7	3125.0/2406.0	0.77	2812.0/956.0	0.34	1.11	No interaction
		6.7	3333.0/2133.0	0.64	4167.0/1750.0	0.42	1.06	No interaction
	LCE + Octyl gallate	5.7	3750.0/2250.0	0.68	25.0/9.8	0.39	1.07	No interaction
		6.7	2500.0/1500.0	0.61	37.5/10.8	0.43	1.04	No interaction
								
*Le. carnosum* ATCC 49367	LCE + Gallic acid	5.7	3750.0/2775.0	0.74	3750.0/1350.0	0.30	1.04	No interaction
		6.7	3333.0/2566.0	0.77	6250.0/2000.0	0.32	1.09	No interaction
	LCE + Octyl gallate	5.7	3750.0/2475.0	0.66	25.0/7.2	0.29	0.95	No interaction
		6.7	2500.0/1875.0	0.75	41.6/11.2	0.27	1.02	No interaction

MIC values (µg/mL) are the mean of at least two experiments in duplicate. MIC_A_ and MIC_B_ are the MIC of two antimicrobial compounds used alone or in combination. FIC = fractional inhibitory concentrations; FICA and FICB are the FIC for compounds A and B, respectively; FICI = fractional inhibitory concentration index. The synergistic effect is defined as FICI ≤ 0.5; no FICI interaction > 0.5–≤4.0; and antagonism as FICI of more than 4.

**Table 3:** Antimicrobial activities of binary combinations of LCE with OG against two strains of LAB in LSM broth at 4 °C for 14 days.

Bacteria Tested	Binary Combination (A + B)	pH	MIC_A_ (Alone/Comb)	FIC_A_	MIC_B_ (Alone/Comb)	FIC_B_	FICI	Interpretation
*C. divergens* ATCC 35677								
	LCE + Octyl gallate	5.7	2500.0/1550.0	0.62	25.0/7.0	0.28	0.90	No interaction
		6.7	5000.0/3400.0	0.69	50.0/15.0	0.30	0.99	No interaction
								
*Le. carnosum* ATCC 49367	LCE + Octyl gallate	5.7	2500.0/1700.0	0.68	12.5/4.4	0.35	1.03	No interaction
		6.7	1250.0/1037.0	0.83	25.0/11.0	0.44	1.27	No interaction

MIC values (μg/mL) are the mean of at least two experiments in duplicate. MIC_A_ and MIC_B_ are the MIC of two antimicrobial compounds used alone or in combination. FIC = fractional inhibitory concentrations; FICA and FICB are the FIC for compounds A and B, respectively; FICI = fractional inhibitory concentration index. The synergistic effect is defined as FICI ≤ 0.5; no interaction FICI > 0.5–≤4.0; and antagonism as FICI of more than 4.

**Table 4:** Change in viable counts (log_10_ cfu/mL) of the two strains of LAB after incubation in LSM broth.

Strain	Growth Conditions	Log ∆ (log_10_ cfu/mL) ^a^		Differences Log ∆ ^b^	
		pH 5.7	pH 6.7	pH 5.7	pH 6.7
*C. divergens* ATTC 35677	LSM	3.13	3.66		
	+GA (MIC)	0.65	2.26		
	+GA (2MIC)	0.88	0.84		
	+OG (MIC)	1.09	3.73		
	+OG (2MIC)	−1.70	0.62		
	+LCE (MIC)	1.85	2.16		
	+LCE (2MIC)	−0.61	−0.28		
	+GA (MIC) + LCE (MIC)	−0.42	−1.39	**−1.07**	**−3.64**
	+GA (2MIC) + LCE (2MIC)	−3.72	−3.40	**−3.13**	**−3.68**
	+OG (MIC) + LCE (MIC)	−0.68	−0.36	**−0.77**	**−0.98**
	+OG (MIC) + LCE (2MIC)	−3.72	−3.40	**−2.02**	**−2.92**
*Le. carnosum* ATCC 49367	LSM	2.01	3.61		
	+GA (MIC)	−1.75	1.03		
	+GA (2MIC)	−2.93	−2.87		
	+OG (MIC)	−3.86	−1.38		
	+OG (2MIC)	−5.03	−2.87		
	+LCE (MIC)	−0.13	1.93		
	+LCE (2MIC)	−2.02	0.18		
	+GA (MIC) + LCE (MIC)	−5.03	−2.87	**−3.28**	**−3.9**
	+GA (2MIC) + LCE (2MIC)	−5.03	−2.87	**−2.10**	**0.00**
	+OG (MIC) + LCE (MIC)	−5.03	−2.87	**−1.17**	**−1.49**
	+OG (MIC) + LCE (2MIC)	−5.03	−2.87	**0.00**	**0.00**

Initial inoculum was 1 × 10^5^–2 × 10^6^ cfu/mL. ^a^ Log Δ is defined as follows: columns with numbers not in bold: final inoculum—initial inoculum (log_10_ cfu/mL). ^b^ Columns with numbers in bold: log Δ for two antimicrobial combination—log Δ of the most active single antimicrobial.

**Table 5:** Change in viable counts (log_10_ cfu/mL) of two strains of LAB after incubation in LSM broth for 14 days at 4 °C.

Strain	Growth Conditions	Log ∆ (log_10_ cfu/mL) ^a^		Differences Log ∆ ^b^	
		pH 5.7	pH 6.7	pH 5.7	pH 6.7
*C. divergens* ATTC 35677	LSM	2.61	2.73		
	+OG (MIC)	−0.95	−2.57		
	+OG (2MIC)	−1.61	−5.02		
	+LCE (MIC)	−0.27	1.19		
	+LCE (2MIC)	−1.49	−0.33		
	+OG (MIC) + LCE (MIC)	−1.40	−5.02	**−0.45**	**−2.45**
	+OG (MIC) + LCE (2MIC)	−4.90	−5.02	**−3.29**	**0.00**
*Le. carnosum* ATCC 49367	LSM				
	+OG (MIC)	1.23	−0.39		
	+OG (2MIC)	−0.14	−3.34		
	+LCE (MIC)	3.88	3.87		
	+LCE (2MIC)	2.12	3.21		
	+OG (MIC) + LCE (MIC)	−0.33	−3.34	**−1.56**	**−2.95**
	+OG (MIC) + LCE (2MIC)	−3.04	−3.34	**−2.90**	**0.00**

Initial inoculum was 1 × 10^5^–4 × 10^6^ cfu/mL. ^a^ Log Δ is defined as follows: columns with numbers not in bold: final inoculum—initial inoculum (log_10_ cfu/mL). ^b^ Columns with numbers in bold: log Δ for two antimicrobial combinations—log Δ of the most active single antimicrobial.

**Table 6:** Viable counts (log_10_ cfu/mL) of microbial groups in poultry fillets under different treatments and days of storage under refrigeration.

Microorganism Groups	Atmosphere	Treatment	Days of Storage				
			0	2	4	6	9
PSC	NA	None	5.16 ± 0.24 ^aA^	6.22 ± 0.35 ^bA^	6.50 ± 0.31 ^bcA^	6.96 ± 0.43 ^cA^	10.22 ± 0.25 ^dA^
		+OG + LCE	4.38 ± 0.27 ^aB^	6.36 ± 0.17 ^bA^	6.54 ± 0.30 ^bA^	7.25 ± 0.25 ^cA^	9.78 ± 0.03 ^dA^
	MA	None	5.16 ± 0.24 ^abA^	4.98 ± 0.34 ^aB^	5.73 ± 0.16 ^bcB^	5.54 ± 0.55 ^cB^	5.99 ± 0.14 ^dB^
		+OG + LCE	4.50 ± 0.32 ^aB^	5.20 ± 0.19 ^bB^	5.83 ± 0.18 ^cB^	5.99 ± 0.14 ^cB^	6.85 ± 0.74 ^dC^
PSEUDO	NA	None	3.27 ± 0.32 ^aA^	3.99 ± 0.31 ^abA^	3.72 ± 1.18 ^aA^	5.22 ± 0.25 ^bA^	7.15 ± 0.20 ^cA^
		+OG + LCE	3.54 ± 0.14 ^aA^	4.40 ± 1.20 ^abA^	4.26 ± 0.50 ^abA^	5.22 ± 0.17 ^bA^	7.02 ± 0.17 ^cA^
	MA	None	3.27 ± 0.32 ^aA^	1.60 ± 0.66 ^bB^	3.13 ± 0.67 ^aA^	3.12 ± 1.07 ^aB^	3.15 ± 0.17 ^aB^
		+OG + LCE	3.39 ± 0.32 ^aA^	2.74 ± 1.20 ^aAB^	3.18 ± 0.64 ^aA^	2.92 ± 0.92 ^aB^	3.39 ± 0.45 ^aB^
VRBGA	NA	None	2.00 ± 0.00 ^aA^	3.32 ± 0.19 ^bA^	2.30 ± 0.42 ^aA^	2.57 ± 0.48 ^aA^	4.64 ± 0.18 ^cA^
		+OG + LCE	1.85 ± 0.17 ^aA^	3.26 ± 0.03 ^bA^	2.77 ± 0.27 ^bA^	3.22 ± 0.49 ^bB^	4.55 ± 0.13 ^cA^
	MA	None	2.00 ± 0.00 ^aA^	2.29 ± 0.40 ^aB^	2.30 ± 0.69 ^aA^	2.35 ± 0.31 ^aA^	2.02 ± 0.44 ^aB^
		+OG + LCE	2.00 ± 0.00 ^aA^	2.93 ± 0.34 ^bA^	2.30 ± 0.43 ^aA^	2.45 ± 0.35 ^abA^	2.54 ± 0.18 ^abB^
PCA	NA	None	4.84 ± 0.25 ^aA^	5.00 ± 0.45 ^aA^	6.28 ± 0.90 ^bA^	6.50 ± 0.34 ^bBC^	9.77 ± 0.13 ^cA^
		+OG + LCE	4.92 ± 0.27 ^abA^	4.28 ± 0.00 ^aAB^	5.57 ± 0.22 ^bAB^	7.20 ± 0.66 ^cC^	9.51 ± 0.03 ^dA^
	MA	None	4.84 ± 0.25 ^abA^	4.16 ± 0.68 ^aAB^	4.53 ± 1.06 ^aB^	5.74 ± 0.49 ^bcAB^	6.19 ± 0.48 ^cC^
		+OG + LCE	4.92 ± 0.27 ^aA^	3.50 ± 0.58 ^bB^	6.14 ± 0.38 ^cA^	5.05 ± 0.36 ^aA^	6.94 ± 0.32 ^dB^
Coliforms	NA	None	2.00 ± 0.00 ^aA^	1.39 ± 0.27 ^bA^	1.78 ± 0.15 ^aA^	0.85 ± 0.17 ^cA^	0.74 ± 0.10 ^cA^
		+OG + LCE	2.00 ± 0.00 ^abA^	1.30 ± 0.30 ^bcA^	2.16 ± 0.55 ^aA^	1.14 ± 0.48 ^cA^	0.78 ± 0.14 ^cA^
	MA	None	2.00 ± 0.00 ^aA^	1.20 ± 0.17 ^bA^	1.78 ± 0.15 ^aA^	0.70 ± 0.00 ^bA^	0.90 ± 0.36 ^bA^
		+OG + LCE	2.08 ± 0.15 ^abA^	1.54 ± 0.28 ^aA^	2.22 ± 0.60 ^bA^	1.83 ± 0.29 ^abB^	1.66 ± 0.12 ^abB^
MRS	NA	None	5.00 ± 0.00 ^aA^	4.05 ± 0.30 ^aA^	5.07 ± 0.56 ^abAB^	4.86 ± 0.24 ^aA^	6.40 ± 0.54 ^aC^
		+OG + LCE	5.00 ± 0.00 ^aA^	3.91 ± 0.21 ^aA^	5.14 ± 0.74 ^abAB^	4.49 ± 0.40 ^aA^	6.23 ± 0.18 ^aC^
	MA	None	5.00 ± 0.00 ^aA^	3.96 ± 0.13 ^aA^	6.01 ± 0.18 ^aB^	4.30 ± 0.54 ^aA^	4.73 ± 0.20 ^bA^
		+OG + LCE	5.00 ± 0.00 ^aA^	3.96 ± 0.13 ^aA^	4.44 ± 0.13 ^bA^	4.50 ± 0.28 ^aA^	5.61 ± 0.17 ^cB^
TSC	NA	None	1.00 ± 0.00 ^abA^	1.56 ± 0.40 ^bcA^	0.70 ± 0.00 ^aA^	1.84 ± 0.53 ^cB^	0.90 ± 0.39 ^abA^
		+OG + LCE	1.00 ± 0.24 ^abA^	1.36 ± 0.24 ^aAB^	0.70 ± 0.00 ^bA^	1.22 ± 0.15 ^abAB^	1.09 ± 0.45 ^abA^
	MA	None	0.85 ± 0.17 ^aA^	0.70 ± 0.00 ^aB^	0.70 ± 0.00 ^aA^	0.92 ± 0.29 ^aA^	0.70 ± 0.00 ^aA^
		+OG + LCE	0.92 ± 0.15 ^abA^	1.14 ± 0.52 ^abAB^	0.70 ± 0.00 ^aA^	1.38 ± 0.15 ^bAB^	0.70 ± 0.00 ^aA^
BP	NA	None	2.27 ± 0.32 ^aA^	3.59 ± 0.39 ^bA^	3.48 ± 0.22 ^bA^	4.79 ± 0.55 ^cA^	3.18 ± 0.35 ^bA^
		+OG + LCE	2.34 ± 0.43 ^aA^	3.25 ± 0.31 ^bcA^	3.10 ± 0.41 ^abcA^	3.86 ± 0.61 ^cA^	2.74 ± 0.09 ^abA^
	MA	None	2.27 ± 0.32 ^aA^	1.92 ± 0.15 ^aB^	1.85 ± 0.30 ^aB^	2.34 ± 0.47 ^aB^	2.07 ± 0.15 ^aB^
		+OG + LCE	2.34 ± 0.43 ^aA^	2.20 ± 0.24 ^aB^	2.13 ± 0.67 ^aB^	2.39 ± 0.48 ^aB^	2.27 ± 0.20 ^aB^

BP: *Staphylococcus* spp.; MRS: lactic acid bacteria; PCA: aerobic plate count; PSC: psychrotrophic bacteria; PSEUDO: *Pseudomonas*, TSC: sulphite-reducing clostridia. MA: modified atmosphere; NA: normal atmosphere. OG: octyl gallate. Values are the mean of at least two experiments in duplicate ± standard deviation. Values with different lowercase letters in the same row indicate significant differences (*p* < 0.05) among days of storage and data with different capital letters in the same column for each microorganism group indicate significant differences (*p* < 0.05) among the four conditions (atmosphere and treatments).

**Table 7:** Factor loading (varimax Kaiser normalized).

	Component	
	1	2
Psychotropic bacteria (PSC)	**0.949**	0.226
*Pseudomonas* (PSEUDO)	**0.937**	0.130
*Enterobactereaceae* (VRBGA)	**0.911**	−0.129
Aerobic plate count (PCA)	**0.869**	0.389
Coliforms	**−0.696**	0.405
Lactic acid bacteria MRSA	0.378	**0.823**
Clostridia sulphite reducing (TSC)	0.334	**−0.700**
*Sthaphylococcus* spp. (BP)	−0.104	−0.595

Values in bold font are the variables that explain the principal component.

## Data Availability

The original contributions presented in the study are included in the article, further inquiries can be directed to the corresponding author.

## Abbreviations

FIC, fractional inhibitory concentration; FICI, FIC index; GA, gallic acid; LCE, *Lippia citriodora* extract; MA, modified atmosphere; MBC, minimal bactericidal concentration; MIC, minimal inhibitory concentration; NA, normal atmosphere; OG, octyl gallate.

## Appendix A

CHROMATOGRAPHIC ANALYSIS OF PLX (Monteloeder, SL., Elche, Spain)

HPLC conditions:

Column: BDS Hypersil C18 250 × 4.6 mm, 5 µm.

Flow rate: 1 mL/min.

Temperature: 30 °C.

Injection volume: 20 µL.

Detector (UV): 330 nm.

Wavelength scans from 190 nm to 500 nm.

Mobile phase A: Water + Acetic acid 2.5%.

Mobile phase B: Acetonitrile.

**Time**	**Mobile Phase A**	**Mobile Phase B**
0	95	5
20	70	30
30	10	90
35	95	5
40	95	5

Dissolutions preparation:

Blank: Water: Methanol (1:1).

Reference Std solution: Verbascoside (Extrasynthese Primary Std): 0.5 mg/mL Water: Methanol (1:1).

Sample solution: 40 mg/25 mL Water: Methanol (1:1).

*HPLC-MS Analysis (Laboratory for Instrumental Techniques, University of León, León, Spain)*

Sample preparation: We prepared a 5% solution of LCE in methanol (*w*/*v*), stirred at 120 rpm at 25 °C for 24 h, and then filtered the solution through a 20 µm diameter filter. The sample was analysed by diluting 5 μL (1/10) in 100 mL MeOH. This dilution was analysed by HPLC-MS.

The HPLC equipment was a Bruker Elute UHPLC, and the mass spectrometer was a TOF time-of-flight mass spectrometer with ion mobility (although mobility has not been used…) called “tims-TOF” from Bruker.

Chromatographic method:

The conditions of the mass spectrometer were:

EndPlate offset	500	V
Capillary	3000	V
Nebulizer	3.0	Bar
Dry Gas	8.0	L/min
DryTemp	220	°C

The scan range was from 50 to 1000 *m*/*z*.

Yellow is the fraction A (phase A: formic acid 0.1% *v*/*v* in water) in the isocratic elution; green is the fraction B (phase B: acetonitrile + formic acid 0.1% *v*/*v*) in the gradient elution.
